# Cationic Nanomaterials for Autoimmune Diseases Therapy

**DOI:** 10.3389/fphar.2021.762362

**Published:** 2022-01-21

**Authors:** Baozhao Xie, Keqian Du, Fujian Huang, Zhiming Lin, Linping Wu

**Affiliations:** ^1^ Division of Rheumatology, Department of Internal Medicine, the 7th Affiliated Hospital, Guang Xi Medical University, Wuzhou, China; ^2^ Department of Rheumatology, Third Affiliated Hospital of Sun Yat-Sen University, Guangzhou, China; ^3^ Center for Chemical Biology and Drug Discovery, Guangzhou Institute of Biomedicine and Health, Chinese Academy of Sciences, Guangzhou, China

**Keywords:** nanomaterials, cationic polymer, autoimmune diseases, nanoparticles, cell-free DNA

## Abstract

Cationic nanomaterials are defined as nanoscale structures smaller than 100 nm bearing positive charges. They have been investigated to apply to many aspects including clinical diagnosis, gene delivery, drug delivery, and tissue engineering for years. Recently, a novel concept has been made to use cationic nanomaterials as cell-free nucleic acid scavengers and inhibits the inflammatory responses in autoimmune diseases. Here, we highlighted different types of cationic materials which have the potential for autoimmune disease treatment and reviewed the strategy for autoimmune diseases therapy based on cationic nanoparticles. This review will also demonstrate the challenges and possible solutions that are encountered during the development of cationic materials-based therapeutics for autoimmune diseases.

## Introduction

Nanomaterials with particles size smaller than 100 nm and bearing positive charges or synthesized in the presence of novel cationic entities, incorporated on their backbone and/or as side chains, are considered as cationic nanomaterials (Rezaei et al., 2019). Cationic nanomaterials are generally divided into two categories: natural or synthetic (Samal et al., 2012). Poly (amidoamine) (PAMAM), polyphosphoramidate (PPA), poly [2-(N,N-dimethylamino) ethyl methacrylate] (PDMAEMA), hexadimethrine bromide (HBMBr) and β-cyclodextrin-containing polycation (CDP) are widely studied among them. For their inherent bioactive properties such as antimicrobial, stimuli responsiveness, antioxidant, antitumor, and anti-inflammatory, cationic polymers are expected to possess further enhanced therapeutic potential (Samal et al., 2012). Furthermore, the unique features of cationic nanomaterials such as desirable size, greater solubility, easier to pass through cellular barriers, and more reactivity make them become attractive options for therapeutic applications (Yonezawa et al., 2020).

Autoimmune diseases are defined as a clinical syndrome caused by the activation of T cells or a loss of B-cell tolerance to particular antigens without infection or other discernible causes (Davidson and Diamond, 2001). Autoimmune diseases vary greatly and are complicated in clinical manifestations, with some appear to be systemic such as systemic lupus erythematosus, some are limited to organ-specific like type 1 diabetes mellitus (Rosenblum et al., 2015). Autoimmunity is initiated by a combination of genetic predisposition and environmental triggers and followed by epitopes spread and inflammatory loop give rise to a vicious cycle (Davidson and Diamond 2001; Rosenblum et al., 2015). Nowadays, disease-modifying anti-rheumatic drugs, glucocorticoids, analgetics, non-steroidal anti-inflammatory drugs, and biological agents are the primary therapeutic method in autoimmune diseases, but the drugs used to suppress the immune response have numerous side effects with large doses and continuous therapy is not conducive to long-term host survival (Miller et al., 2007). Hence, searching for novel therapeutic methods is crucial.

Recently, with the advancement of our understanding of nanotechnology, nanomaterials have become a promising approach for the treatment of autoimmune diseases. Cationic nanomaterials have become one of the important pillars of nanomaterials. The therapeutic applications of cationic nanomaterials mainly focus on three aspects: gene delivery, drug delivery and tissue engineering (Samal et al., 2012). Recently, successful attempts have been reported that cationic nanomaterials possessed the therapeutic potential severed as drugs. In this review, we highlight progress on the therapeutic potential of cationic nanomaterials in autoimmune diseases, including rheumatoid arthritis (RA), systemic lupus erythematosus (SLE), autoimmune skin inflammation, and discuss the dilemma of cationic nanomaterials in the therapy of autoimmune diseases.

## Cationic Materials

### Poly (amidoamine) (PAMAM)

**FIGURE 1:**
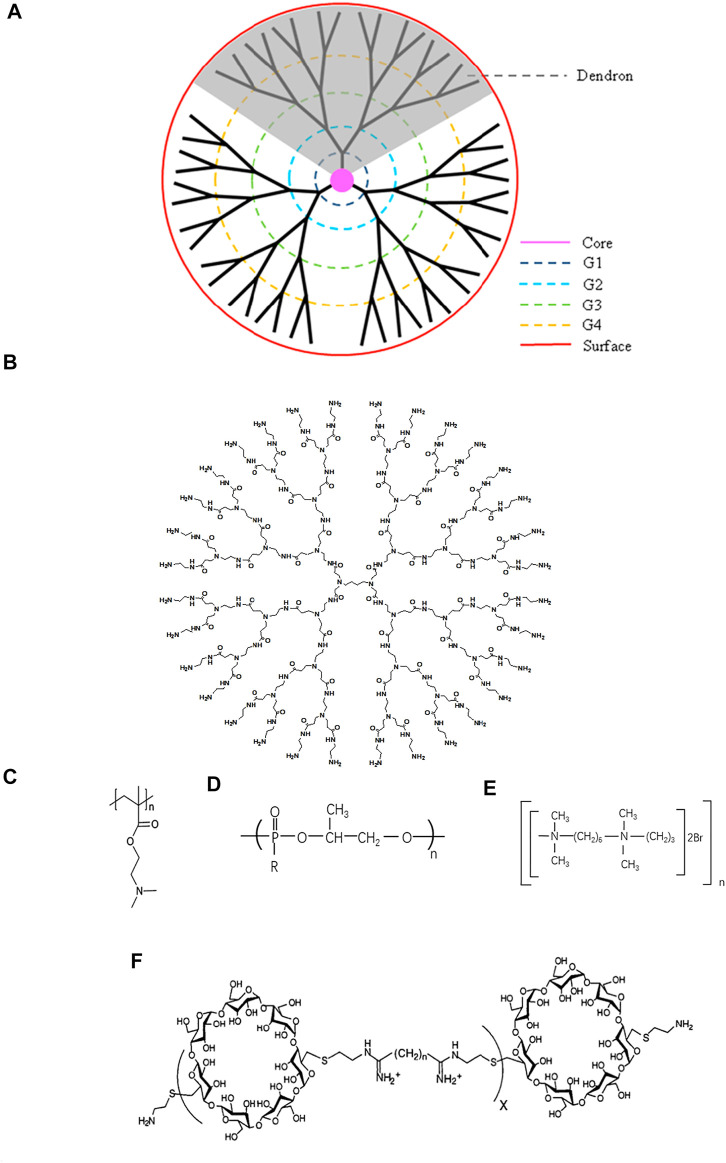
Schematic structure of typical cationic polymers. **(A)** Dendrimer (Reprint from Reference (Wu et al., 2015)). **(B)** Genearlation 3 poly (amidoamine) (PAMAM). **(C)** Poly (2-(diethylamino) ethyl methacrylate) (PDMA) (Reprint from Reference (Samal et al., 2012). **(D)** Polyphosphoramidate (PPA) (Reprint from Reference (Zhang PC. et al, 2005)). **(E)** Hexadimethrine bromide (HBMBr) (Reprint from Ref(Aubin et al., 1997)). **(F)** β-cyclodextrin-containing polycation (CDP) (Reprint from Ref (Hwang et al., 2001)).

The dendrimers were first synthesized by Tomalia et al. taking advantage of the architecture including monodispersity, extraordinary symmetry, hyper branch with tree-like structures (Tomalia et al., 1985). They consist of a central core and branches emanating from the core terminated with functional surface groups (Figure 1B). With the increase of generation, dendrimers form 3D spheres, thus creating supramolecular void spaces that can bind and transfer other molecules (Quadir and Haag 2012; Dzmitruk et al., 2018). In addition, it has multiple surface functional groups that can be modified, which is different from linear structures (Menjoge et al., 2010). A study had shown that linear structure had stronger DNA binding and cellular uptake, but dendritic structure mediated gene expression is higher than linear structure, which may be related to the escape of sufficient DNA amount of effective gene expression into the cytoplasm (Yamagata et al., 2007).

PAMAM is one of the most widely studied dendrimers. Because of the ability to combine with nucleic acid, numerous effort has been developed in applying PAMAM to the treatment of diseases including gene delivery and nucleic acid scavenge (Abedi-Gaballu et al., 2018). However, the binding efficiency is associated with the positive charge density. Higher generation PAMAM with more primary amines possess higher positive charge density on the surface (Jensen et al., 2011). G3-G10 PAMAM dendrimers are the optimal choices resulted from their remarkable stability to combine with nucleic acid and higher transfect efficiency (Palmerston Mendes et al., 2017). Successful attempts have been made to use PAMAM-G3 as antithrombotic agents and anti-metastatic agents. Due to the property of combination with nucleic acid, PAMAM-G3 attenuated the activation of blood coagulation and inflammation induced with nucleic acid through the Toll-like receptor (TLR) pathway, which was related to the thrombosis, lung metastasis in breast cancer, and liver metastasis in pancreatic cancer (Jain et al., 2012; Naqvi et al., 2018; Holl et al., 2020).

Furthermore, surface modification and tri-block modification have been applied to PAMAM. Compared with polyamidoamine (PAMAM), PEGylation modification of PAMAM could increase the transfection efficiency and stabilization with lower cytotoxicity (Reyes-Reveles et al., 2013; Sun et al., 2014). A novel tri-block nanocarriers consisting of PAMAM, poly(ethylene glycol) (PEG), and poly-l-lysine (PLL) were developed to deliver siRNA. PLL replaced the role of PAMAM to form polyplexes with siRNA and PAMAM severed as a proton sponge. PEG was used to stabilize nanocarriers in plasmas (Patil et al., 2011). Biswas et al. also developed another triblock nanocarrier PAMAM-G4-D-PEG-DOPE to deliver siRNA. Different from the former, PAMAM-G4 worked for efficient siRNA condensation (Biswas et al., 2013). Similarly, PAMAM, PEG, and lactobionic acid (Gal) were used to construct a delivery system to carry AEG-1 siRNA, and it was proved that PAMAM-AEG-1si nanoplexes restrain tumor growth (Rajasekaran et al., 2015).

### Poly(2-(diethylamino)ethyl Methacrylate) (PDMA)

As an important pH bioresponsive functional cationic nanomaterials, as well as the character of excellent stability and safety profile (Huang et al., 2018), PDMA (Figure 1C) has been widely applied as a delivery system. Possessing a high affinity for nucleic acid, PDMA is increasingly studied in the gene delivery and neutralization of nucleic acid. A biodegradable cationic micelles PDMAEMA-PCL-PDMAEMA was established to deliver siRNA and paclitaxel into cancer cells, which diminished the expression of VEGF (Zhu et al., 2010). Rungsardthong et al. demonstrated that the DNA affinity of PDMA could be fine-tuned by varying the PH and the polymer/DNA ratios (Rungsardthong et al., 2003).

Researchers anticipated that the properties of PDMA could be optimized by modifying different groups. Deshpande et al. utilized 2-(dimethylamino) ethyl methacrylate (DMAEMA) to construct three different polymers: DMAEMA-PEG (a diblock copolymer), DMAEMA-OEGMA 7 (a brush-type copolymer), and DMAEMA-stat-PEGMA (a comb-type copolymer). Compared with PDMAEMA, all of them exhibited more excellent binding ability with oligonucleotide while DMAEMA-stat-PEGMA showed the best. But DMAEMA-PEG and DMAEMA-OEGMA 7 own better long-term colloidal stability (Deshpande et al., 2002). PEO-PPO-PEO-pDMAEMA (L92-pDMAEMA) and PEO-pDMAEMA copolymers basing on PDMA, poly (propylene oxide) (PPO), and poly (ethylene oxide) (PEO) were also developed to deliver genes. It has been reported that modification with PEO could reduce the unfavorable interactions with complement factors or cellular components (Bromberg et al., 2005). Furthermore, PEO-b-PDMAEMA could form soluble complexes with DNA of a much smaller size resulted from the amphiphilic nature of the polymer (Tan et al., 2006).

### Polyphosphoramidate (PPA)

Polyphosphoramidate (PPA) (Figure 1D), a biodegradable cationic material, has been investigated as drug delivery and gene delivery for years. Their structures, different side chains, molecular weight, and positive charge on the surface can influence the complexation with nucleic acids. PPA owned higher DNA binding efficiency when molecular weight and positive charge density increased (Ren et al., 2010). Zhang et al. developed a series of cationic polymers which had an identical backbone and different side chains including primary, secondary, tertiary, and quaternary amino groups, and demonstrated that PPA with primary amino group possessed uppermost ability to complex with nucleic acids (Wang et al., 2004). Furthermore, the same team synthesized ternary complexes, consisting of PPA backbone, primary and tertiary amino group, and quaternary complexes, containing PPA, primary, secondary, and tertiary amino groups. And the results showed that the coexistence of primary and other amino groups could elevate the combination with the nucleic acid (Zhang PC. et al, 2005).

PEGylation modification is also applied in PAA. PEG-b-PPA/DNA micelles with lower surface charge and smaller particle size ranging from 80 to 100 nm maintained similar transfection efficiency while showing lower cytokines and better biocompatibility compared with PPA/DNA (Jiang et al., 2007). Moreover, galactosylated PPA was prepared to enhance the targeted capacity as a delivery system. However, the transfection efficiency of gal-PPA reduced with the increase of galactose substitution degree, presumably resulting from the decreased DNA binding capacity and particle stability (Zhang XQ. et al, 2005). Hence, modification of PPA needs to be further explored.

### Hexadimethrine Bromide (HBMBr)

Hexadimethrine bromide (HDMBR) (Figure 1E) has been used as an antiheparin agent for many years (Pai and Crowther 2012) and has been rediscovered in recent years to neutralize nucleic acids and deliver genes due to its positive charge. A combination of HDMBr and dimethyl sulfoxide facilitated DNA transfection into chicken embryo fibroblast cells and human fibroblast (Kawai and Nishizawa 1984; Aubin et al., 1997). After intraperitoneally administered, HDMBR neutralized extracellular nucleic acids and thereby reduced lung injury, restrained disruption of alveolar-capillary barrier, and increased blood oxygenation in acute respiratory distress syndrome (ARDS) model rats exposed to CEES, a toxic chemical (Mariappan et al., 2020). Similarly, HDMBr could scavenge mitochondrial DNA (mtDNA) in an *in vivo* model of trauma hemorrhage, and the ability to inhibit inflammation and apoptotic cell death emerged (Aswani et al., 2018). But a crucial concern was the toxicity of HDMBr, including the nephrotoxicity and neurotoxicity, which may be a critical challenge for its biomedicine application (Pai and Crowther 2012; Bao et al., 2018).

### β-Cyclodextrin-Containing Polycation(CDP)

β-cyclodextrin-containing polycation (CDP) (Figure 1F), a classical cationic compound, is widely studied in gene delivery. The introduction of β-cyclodextrin, which is itself a large carbohydrate, reduced the toxicity of the polymer (Reineke and Davis 2003). A delivery system, consisting of a CDP, a polyethylene glycol (PEG) steric stabilization agent, and human transferrin (Tf) encapsulated ribonucleotide reductase subunit M2 siRNA, was administrated in non-human primates and the result showed that the nanoparticles could be safely used in non-human primates (Heidel et al., 2007). The same team conducted a phase I clinical trial and the nanoparticle indeed diminished the expression of mRNA (Davis et al., 2010).

It’s has been reported that the DNA binding efficiency of CDP was related to the structure. Hwang et al. synthesized five compounds composed of dicysteamine-β-cyclodextrin and other difunctionalized comonomers. And DNA affinity, DNA protective ability, and the toxicity of polymers altered with the number of methylene groups within the difunctionalized comonomers. When the number of methylene groups was six, the spacing between the cationic amidine groups is optimal for DNA binding (Hwang et al., 2001). Besides, maintaining stability *in vivo* is also a concern for CDP. Researchers have found that the introduction of PEG or Adamantane (AD) could enhance the stability of CDP (Heidel 2011).

## Therapeutic Potential in Autoimmune Diseases

**FIGURE 2:**
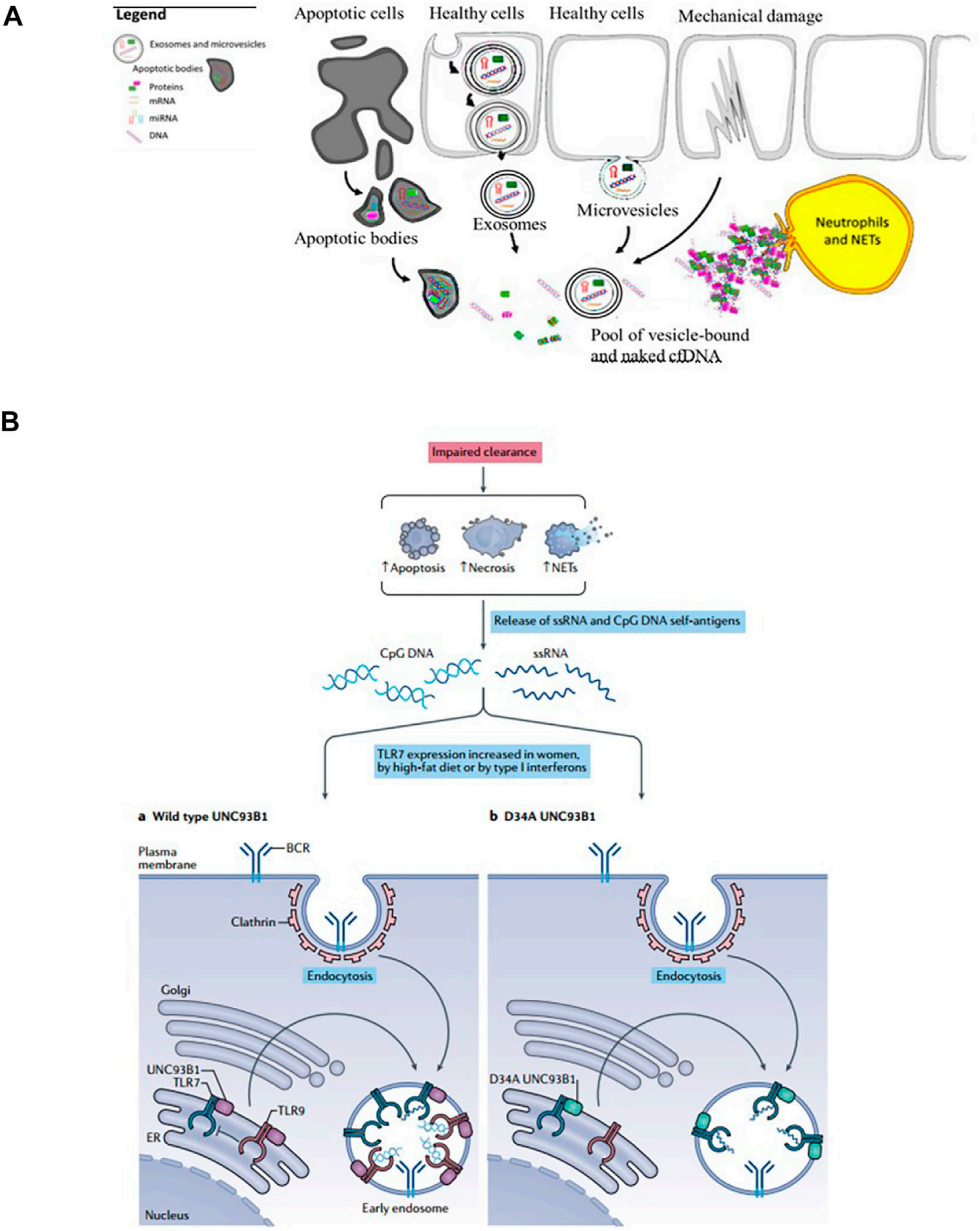
The sources and mechanism of cfDNA. **(A)** Endogenous sources of cfDNA including apoptotic bodies, exosomes, microvesicles, neutrophil extracellular traps (NETs), necrosis (Reprint from Ref (Kubiritova et al., 2019)). **(B)** cfDNA induces inflammatory responses and the occurrence and development of inflammatory diseases (Reprint from Reference (Fillatreau et al., 2021)).

The plasmas cell-free DNA (cf DNA) was first described in 1948 (Mandel and Metais 1948) and the elevated level of cfDNA was observed in patients with rheumatic disease (Tug et al., 2014). Endogenous sources of cfDNA include apoptotic bodies, exosomes, microvesicles, neutrophil extracellular traps (NETs), necrosis (Kubiritova et al., 2019) (Figure 2A). The imbalance of generation and clearance of the cell-free DNA is closely associated with the pathogenesis of autoimmune diseases, such as systemic lupus erythematosus (SLE), rheumatoid arthritis (RA) (Munoz et al., 2010; Dong et al., 2020). If cfDNA is not properly cleared, they can trigger activation of endosomal TLRs such as TLR7, 8, and 9, and thereby induce inflammatory responses (Barrat et al., 2005; Dong et al., 2020; Fillatreau et al., 2021) (Figure 2B).

**FIGURE 3:**
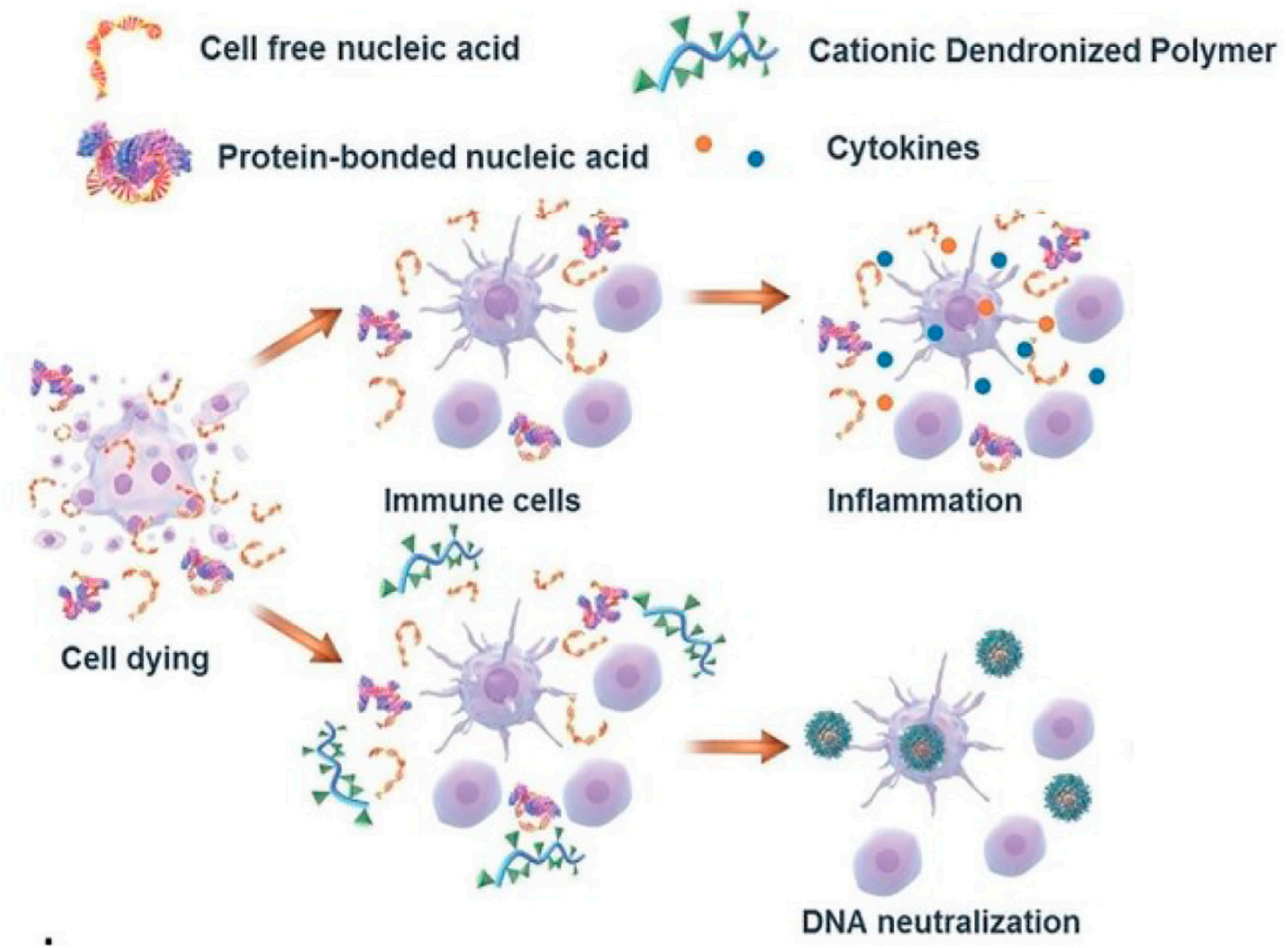
Mechanism of cationic nanomaterials bind nucleic acids to attenuate nucleic acid-mediated activation of immune cells (Reprint from Reference (Peng et al., 2019)).

**FIGURE 4:**
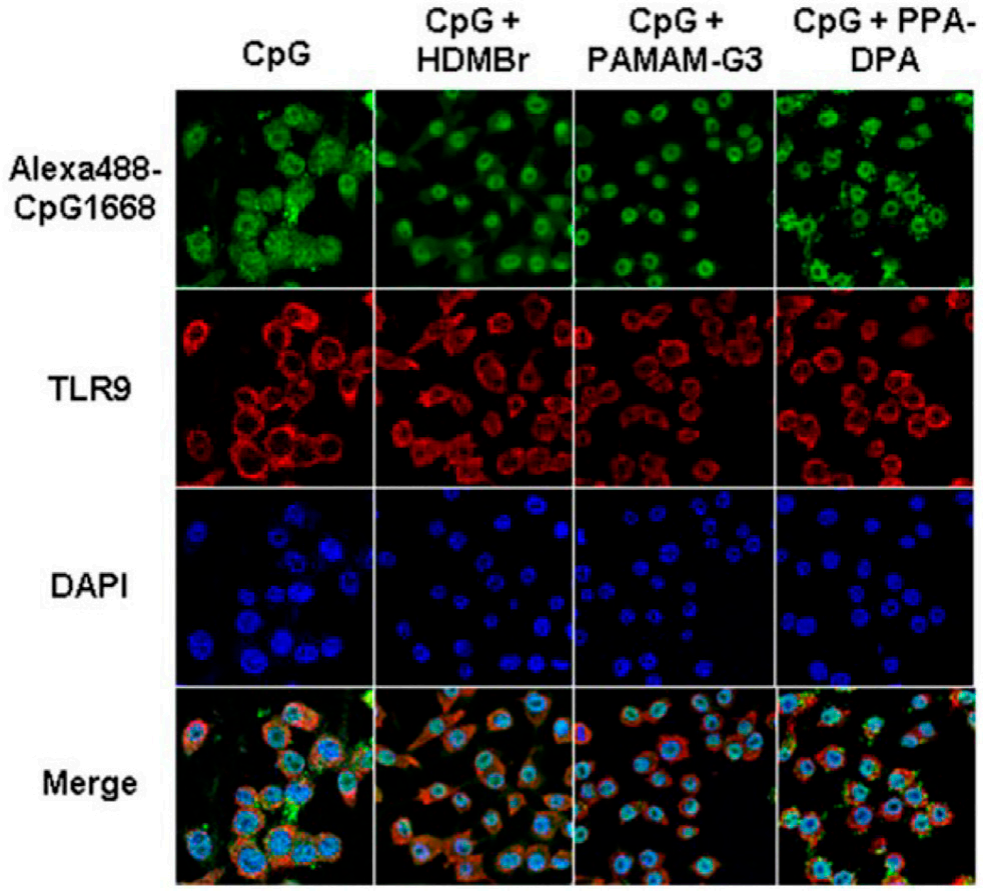
PAMAM-G3, HDMBr, and PPA-DPA altered the uptake and intracellular distribution of immune stimulatory nucleic acids (Reprint from Reference (Lee et al., 2011)).

Due to the capacity of interacting with nucleic acids and forming electrostatic complexes, numerous cationic nanoparticles have been widely used for the non-viral transfection of cells with plasmid DNA, miRNA, and siRNA (Yonezawa et al., 2020). Recently, the interest in exploiting cationic nanomaterials as the nucleic acid-binding polymer to inhibit inflammatory immune diseases emerged. It’s been reported some cationic nanomaterials possess the ability to attenuate nucleic acid-mediated activation of TLRs on macrophages if binding nucleic acids (Figure 3). Sullenger et al. evaluated six of them and found that PAMAM-G3 and HDMBr inhibited the nucleic acid-mediated TLR activation through neutralizing extracellular inflammatory nucleic acids and altering the uptake and intracellular distribution of immune stimulatory nucleic acids (Lee et al., 2011) (Figure 4). Consistently, another research demonstrated that CDP, PAMAM-G3, and HDMBr can inhibit the binding of Lupus anti-DNA antibody and DNA by displacing antibodies from preformed complexes (Stearns et al., 2012). They also observed that nucleic acid scavenging polymers only limited the activation of the immune system by accessible extra-cellular nucleic acid and do not engender non-specific immune suppression (Holl et al., 2013). In some inflammatory diseases such as sepsis, studies had also found that cationic nanomaterials as nucleic acid scavengers could effectively reduce the severity of the disease (Dawulieti et al., 2020; Liu et al., 2021).

For the ability to scavenge nucleic acids, cationic nanomaterials were attempted for the treatment of autoimmune diseases.

### Systemic Lupus Erythematosus (SLE)

SLE is characterized by increased apoptosis and impaired clearance of apoptotic cells. Many factors can influence the clearance of the cell-free DNA in SLE patients, including the abnormalities of DNase activity to clear cf DNA, the combination of the cfDNA with the antibodies, proteins, and nucleosomes, and thus potentially activate inflammatory pathways (Courtney et al., 1999; Duvvuri and Lood 2019). PAMAM-G3 was proved that local administration of it facilitated wound healing in a cutaneous lupus erythematosus (CLE) prone animal by diminishing extracellular nucleic acids and inhibiting TLR7 and TLR9 activation (Holl et al., 2016). Furthermore, researchers also evaluated the ability of PAMAM-G3 to reduce glomerulonephritis and circulating autoantibody levels in MRLlpr mice (Holl et al., 2016).

### Rheumatoid Arthritis

**FIGURE 5:**
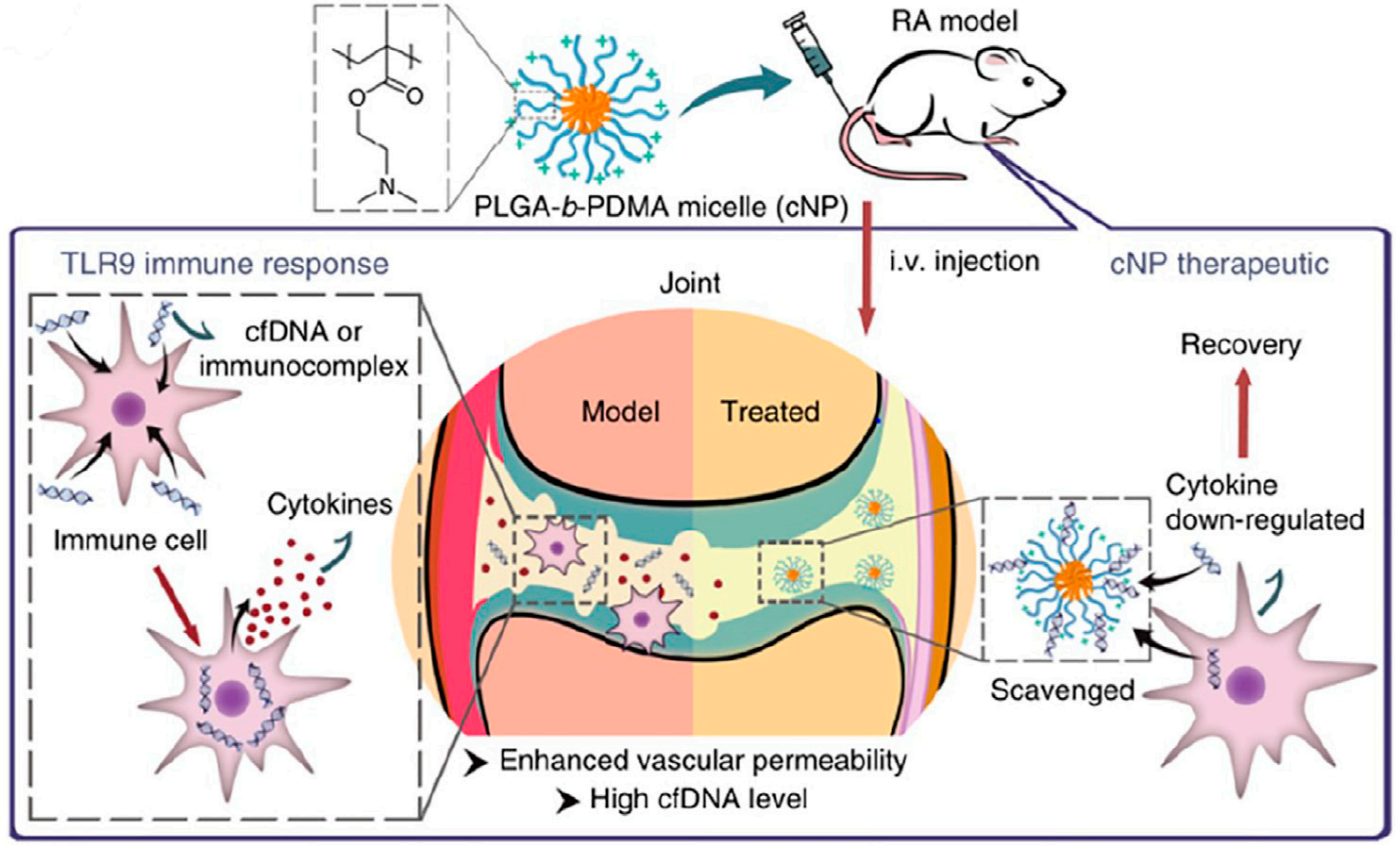
Mechanism of applied cationic nanoparticles to scavenge cfDNA or immunocomplex and thereby prevent the activation of immune cells, down-regulation the expression of cytokines, and alleviate the symptoms of RA (Reprint from Reference (Liang H. et al, 2018)).

The elevated level of cf DNA was discovered in Rheumatoid arthritis patients and whole-genome shotgun sequencing showed SFcfDNAs in RA are enriched with specific CMR sequences, which are hypomethylated (Dong et al., 2020). Therefore, neutralizing cfDNA may be a potential treatment for Rheumatoid arthritis. In a recent study, the researchers prepared a self-assembly PLGA-block-PDMA block copolymer, PLGA-b-PDMA463, and they discovered that it could neutralize cfDNA derived from RA patients and inhibit nucleic acid-mediated activation of primary synovial fluid monocytes and fibroblast-like synoviocytes by restraining the activation of TLR9 (Figure 5). After intravenous injecting PLGA-b-PDMA463 into a CpG-induced mouse model or collagen-induced arthritis rat model (CIA model), successful prevention of RA symptoms, which was evaluated by inflammation, swelling, and deformities of the paws, was achieved and it might be attributed to capacity to scavenge cfDNA and a more favorable biodistribution (Liang H. et al, 2018). With the intent to boost the binding affinity and avoid potential systemic toxicities of PDMA-based cationic nanoparticles (cNPs), the same team tuned the proportion of PLGA and PDMA and introduced poly (ethylene glycol) (PEG) segments to the cNPs’ PDMA shell. The introduction of PEG segments translated into a lower DNA binding efficacy while preserving the ability to hamper joint inflammation. Moreover, due to a greater accumulation and longer retention at the inflamed joints, new NPs were allowed for a lower frequency of administration (Wu JJ. et al, 2020). And another cationic nanoparticle, PCL-g-PAMAM, was also developed to inhibit synovial inflammation and relieve joint inflammation and damage in the CIA mouse model (Peng et al., 2019). Moreover, differences in surface groups of cationic nanomaterials could change their DNA scavenging ability and anti-inflammatory effect in RA, which may be related to different adsorption of opsonin protein. Hydroxylated nanoparticles could prolong the retention in joints and enhance anti-inflammatory effects (Liu. et al., 2021).

### Autoimmune Skin Diseases

**FIGURE 6:**
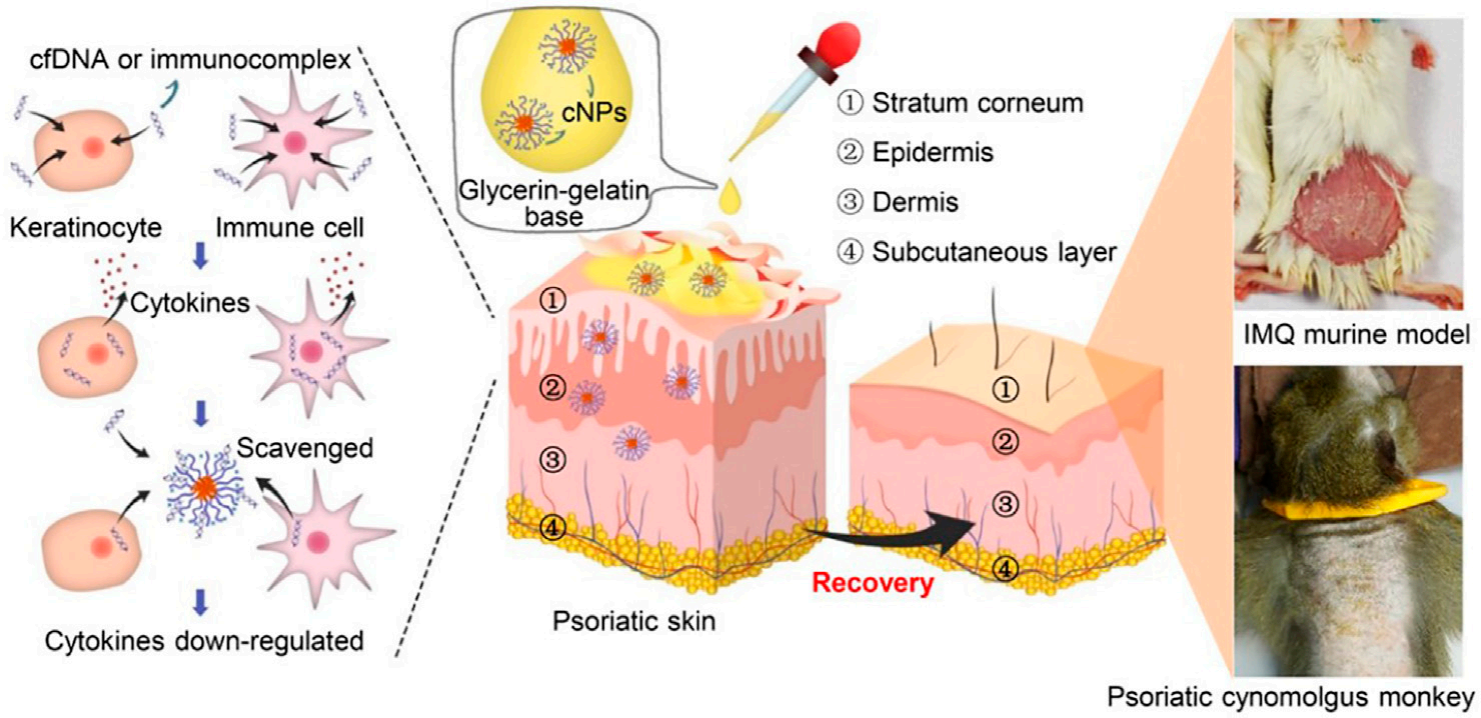
Mechanism of topical administration of PLGA-b-PDMA on psoriasiform skin in both mouse model and cynomolgus monkey model by competing for DNA from the DNA-LL37 immunocomplex and suppressing DNA-LL37-induced cell activation (Reprint from Reference (Liang et al., 2020)).

It’s has been reported that a significantly elevated level of cfDNA in psoriasis patients (Beranek et al., 2017). Topical administration of PLGA-b-PDMA on psoriasiform skin of an IMQ-induced mouse model could alleviate psoriatic symptoms by efficiently competing for DNA from the DNA-LL37 immunocomplex and suppressing DNA-LL37-induced cell activation. Consistent with this result, the application of PLGA-b-PDMA in a cynomolgus monkey model relieved the symptoms of psoriasis (Liang et al., 2020) (Figure 6). A series of cationic materials, poly (2-(dimethylamino) ethyl methacrylate) grafted hairy silica particles (cSPs), with different PDMA lengths and different particle sizes had been studied. These cationic materials also had the ability to scavenge cfDNA and effectively inhibited psoriatic skin inflammation and inflammatory cytokines secretion. In addition, they showed that different particle sizes and the ratio of PDMA affect DNA binding affinity, which was related to anti-inflammatory effects and the ability to enter the dermis (Yan et al., 2021).

### Challenge of Cationic Nanomaterials

The appearance of cationic nanomaterials gives a novel direction in the treatment of disease, which is closely linked to the broad range of properties they offer. However, the safety profiles of cationic nanomaterials have been a critical concern in therapeutic researches (Wu LP. et al, 2020).

### Toxicity

Cytotoxicity, immune-related toxicity, and systemic toxicity are the main barriers to the application of cationic nanomaterials. The application of cationic nanomaterials was restricted due to toxicity such as cell necrosis, inflammatory toxicity, pulmonary toxicity, leukopenia, and thrombocytopenia (Liang X. et al, 2018).

The cytotoxicity of cationic nanomaterials is mainly attributed to the positive charges. Compared with the anion group, cationic nanomaterials exhibit higher cytotoxicity and lethal effects (Calienni et al., 2017; Pereira et al., 2019). Cationic nanomaterials destroy plasma membrane integrity, mitochondrial and lysosomal damage, and more autophagosomes (Mecke et al., 2005; Frohlich 2012). It has been reported that cationic surfactants incorporated into nanoparticles induced cell necrosis and the release of mediators, which resulted from accelerating cell membrane lysis and Ca^2+^ influx *via* the interaction with the cell membrane (Hwang et al., 2015). Another study demonstrated that cytotoxicity decreased in the presence of increases in serum which was based on serum masking of the PEI surface and decrease of the interaction with cell (McConnell et al., 2016). Both of them illustrated that high positive charge density could increase cytotoxicity. A study found that cationic nanomaterials induced cell necrosis rapidly through inhibition of Na^+^/K^+^-ATPase and subsequent leakage of mitochondrial DNA from necrotic cells. Mitochondrial DNA triggered severe inflammation *in vivo* by a pathway involving TLR9 and MyD88 signaling (Wei et al., 2015). But the same team also discovered that the inflammatory response induced by cationic nanocarriers was gradually and spontaneously regressed within 1 week. They hypothesized that cationic nanoparticles negatively regulated inflammation and the result demonstrated that leaked mtDNA altered the phenotype of monocyte via a STING- or TLR9 pathway and PEG2 secreted from Ly6C^+^mancytes inhibited neutrophil activation (Liu et al., 2018).

Similarly, Immunotoxicity limits the application of cationic nanomaterials. Cationic nanomaterials could alter the immune state *via* suppressing innate immunity such as inhibition of natural killer (NK) cell activity, reduction of CD4+/CD8+ ratio, and inflammation cytokines (Kim et al., 2014). Macrophages were also been proved that they could be activated by cationic nanomaterials depending on TLR4 (Toll-like receptor 4) and ROS (reactive oxygen species) signaling (Zhang et al., 2014; Mulens-Arias et al., 2015).

In animal models, the pulmonary toxicity of cationic nanomaterials was also described. Acute lung injury induced by the intratracheal instillation of cationic polyamidoamine dendrimer (PAMAM) nanoparticles had been reported and the model demonstrated cationic nanoparticles suppressed the activity of ACE2 via binding with ACE2, resulting in an imbalance of the renin-angiotensin system (Sun et al., 2015).

Although cationic nanomaterials have not yet entered the stage of clinical research in the treatment of autoimmune diseases, certain systemic toxicity has been found in clinical studies of cationic nanomaterials in tumors. Fatigue, chills, fever, and nausea were mostly described in clinical trials (Zuckerman et al., 2014; Autio et al., 2018). Cardiovascular symptoms such as sinus bradycardia, tachycardia, and hypotension have been reported in a phase Ia/Ib clinical data with polymer-based nanoparticle containing siRNA and a Phase I study of systemically delivering p53 nanoparticle in advanced solid tumors, respectively (Senzer et al., 2013; Zuckerman et al., 2014). In a phase I study for advanced solid tumors, patients experienced infusion-related hypersensitivity which could have been controlled by the frequency after pretreatment with drugs (Rudin et al., 2004). Besides, hematologic disorders including thrombocytopenia and lymphocytopenia occurred in the clinical trials of cationic nanomaterials (Rudin et al., 2004; Zuckerman et al., 2014).

### Strategies to Minimize Toxicity

To minimize the toxicity, two strategies have been presented: designing and synthesizing biodegradable nanomaterials or masking of peripheral charge of nanomaterials by surface engineering (Jain et al., 2010).

It is generally accepted that poly (d,l-lactide-co-glycolide) (PLGA), taking advantage of remarkable biocompatibility, biodegradability, solubility, and stability, plays a pivotal role as delivery systems for drug and gene, scaffold in tissue engineer and drug in treatment. Aragao-Santiago et al. compared the toxicity of biodegradable and non-biodegradable nanoparticles *via* nebulization and discovered that biodegradable PLGA mostly accumulated in lung and eliminated to half in 17.5 to 19.9 h without an elevated level of IL-6 and TNF-α in bronchoalveolar lavage (BAL) supernatant while non-biodegradable nanoparticle induced overexpression of pro-inflammation cytokines and the recruitment of polymorphonuclear to BAL (Aragao-Santiago et al., 2016). Sun et al. constructed a biodegradable micellar nanoparticle consisting of monomethoxy poly (ethylene glycol), poly (epsilon-caprolactone) (PCL), and poly (2-aminoethyl ethylene phosphate) to deliver siRNA and the nanoparticles showed non-toxicity even at high concentrations (Sun et al., 2008). Biocompatible and biodegradable polymers provide non-toxic building blocks for the treatment of diseases, such as PLGA, PLA, PCL we have mentioned above (Hu et al., 2014). The toxicity of cationic materials can be effectively alleviated by introducing these groups.

Zhang et al. synthesized a series of terpolymer with low charge density and high molecular weight, which possessed low toxicity and high conversion efficiency (Zhou et al., 2011). Consistent with this finding, another study illustrated that the toxicity decreased with the increase of particle size (Yan et al., 2021). Using high molecular weight and increased hydrophobicity to compensate for low charge density may be a good strategy to balance performance and toxicity (Mastrobattista and Hennink 2011).

Another strategy for the reduction of toxicity is the modification of the nanoparticle surface. In addition to this, modification of nanomaterial surface possesses extra properties such as prolongation of the retention time, improvement of biodistribution and efficiency, and so on (Jain et al., 2010). Polyethylene glycol (PEG) is the most widely used to coat cationic nanomaterials. Karabasz et al. confirmed that five-layer positively charged poly-l-lysine-terminated nanocapsules (NC5) with rapid hematotoxicity did not show cytotoxicity after being incorporated with PEG (Karabasz et al., 2018). PEG could invest in cationic nanomaterials stealthiness without inducing blood, kidney, spleen, and liver acute and extended acute toxicity (Perret et al., 2018). However, it has been reported that modification of nanomaterials with PEG could trigger activation of both the complement and coagulation systems (Pham et al., 2011). But Pannuzzo et al. put forward the solution. They modified nanomaterials with appropriate combinations and proportions of carboxyPEG2000 and methoxyPEG550 can and indeed inhibited activation of complement (Pannuzzo et al., 2020). Besides, other polymers have gradually been developed as modifications to cationic materials such as poly [N-(2-hydroxypropyl)methacrylamide], poly (carboxybetaine), poly (hydroxyethyl-l-asparagine), or poly-l-glutamic acid (Hu et al., 2014). Toy et al. modified primary amines with imidazole-acetic-acid (IAA) to secondary and tertiary amines and demonstrated that introduction of IAA could abate toxicity and immunotoxicity from branched polyethylenimine (bPEI) and chiton through the TLR4 pathway (Toy et al., 2019). A biodegradable, polyelectrolyte m ultilayer shell consisting of poly-l-lysine (PLL) and poly-L-glutamic (PGA) acid was coated with PGA(NC-PGA) and PEG (NC-PEG), respectively. The biochemical and histopathological evaluation suggested that neither of them showed acute or chronic hematotoxicity, hepatotoxicity, or nephrotoxicity. Compared with NC-PEG, NA-PGA didn’t provoke activation of immune system (Karabasz et al., 2019).

## Conclusion

In conclusion, extracellular nucleic acid is an important trigger mechanism in the development and progression of autoimmune diseases, and scavenging extracellular nucleic acid may be one of the candidates attempt to suppress the occurrence and severity of autoimmune diseases. However, the relevant research is still deficient. The use of cationic compounds, scavengers of extracellular nucleic acids, is only in its infancy as a novel treatment for autoimmune diseases. The applied cationic materials are concentrated on the several materials mentioned in the article, but more potential materials were not be studied. More research in the future can focus on other materials, including structural improvements and proportion optimization. At present, cationic materials have been thoroughly studied in various fields, and clinical studies have been carried out on some drugs and gene delivery. Despite the current challenges in cationic nanomaterials, continued improvements will likely yield achieving the new balance between low toxicity and high therapeutic efficacy *in vivo*, which enormous effort need to be devoted to. In addition, the structure construction, proportion distribution, dosage, usage, and pharmacokinetics of cationic compounds also need further exploration. The overall development prospect is considerable.

## References

[B1] Abedi-Gaballu F. Dehghan G. Ghaffari M. Yekta R. Abbaspour-Ravasjani S. Baradaran B. (2018). PAMAM Dendrimers as Efficient Drug and Gene Delivery Nanosystems for Cancer Therapy. Appl. Mater. Today 12, 177–190. 10.1016/j.apmt.2018.05.002 30511014 PMC6269116

[B2] Aragao-Santiago L. Hillaireau H. Grabowski N. Mura S. Nascimento T. L. Dufort S. (2016). Compared *In Vivo* Toxicity in Mice of Lung Delivered Biodegradable and Non-biodegradable Nanoparticles. Nanotoxicology 10, 292–302. 10.3109/17435390.2015.1054908 26573338

[B3] Aswani A. Manson J. Itagaki K. Chiazza F. Collino M. Wupeng W. L. (2018). Scavenging Circulating Mitochondrial DNA as a Potential Therapeutic Option for Multiple Organ Dysfunction in Trauma Hemorrhage. Front. Immunol. 9, 891. 10.3389/fimmu.2018.00891 29867926 PMC5951958

[B4] Aubin R. A. Weinfeld M. Taghavi M. Mirzayans R. Paterson M. C. (1997). Highly Effective Delivery of Foreign DNA to Adherent Cells via polybrene/DMSO-Assisted Gene Transfer. Methods Mol. Biol. 62, 319–342. 10.1385/0-89603-480-1:319 9108531

[B5] Autio K. A. Dreicer R. Anderson J. Garcia J. A. Alva A. Hart L. L. (2018). Safety and Efficacy of BIND-014, a Docetaxel Nanoparticle Targeting Prostate-specific Membrane Antigen for Patients with Metastatic Castration-Resistant Prostate Cancer: A Phase 2 Clinical Trial. JAMA Oncol. 4, 1344–1351. 10.1001/jamaoncol.2018.2168 29978216 PMC6233779

[B6] Bao F. Shi H. Gao M. Yang L. Zhou L. Zhao Q. (2018). Polybrene Induces Neural Degeneration by Bidirectional Ca2+ Influx-dependent Mitochondrial and ER-Mitochondrial Dynamics. Cell Death Dis 9, 966. 10.1038/s41419-018-1009-8 30237514 PMC6148003

[B7] Barrat F. J. Meeker T. Gregorio J. Chan J. H. Uematsu S. Akira S. (2005). Nucleic Acids of Mammalian Origin Can Act as Endogenous Ligands for Toll-like Receptors and May Promote Systemic Lupus Erythematosus. J. Exp. Med. 202, 1131–1139. 10.1084/jem.20050914 16230478 PMC2213213

[B8] Beranek M. Fiala Z. Kremlacek J. Andrys C. Krejsek J. Hamakova K. (2017). Changes in Circulating Cell-free DNA and Nucleosomes in Patients with Exacerbated Psoriasis. Arch. Dermatol. Res. 309, 815–821. 10.1007/s00403-017-1785-5 29027583

[B9] Biswas S. Deshpande P. P. Navarro G. Dodwadkar N. S. Torchilin V. P. (2013). Lipid Modified Triblock PAMAM-Based Nanocarriers for siRNA Drug Co-delivery. Biomaterials 34, 1289–1301. 10.1016/j.biomaterials.2012.10.024 23137395 PMC3511617

[B10] Bromberg L. Deshmukh S. Temchenko M. Iourtchenko L. Alakhov V. Alvarez-Lorenzo C. (2005). Polycationic Block Copolymers of Poly(ethylene Oxide) and Poly(propylene Oxide) for Cell Transfection. Bioconjug. Chem. 16, 626–633. 10.1021/bc049749f 15898731

[B11] Calienni M. N. Feas D. A. Igartúa D. E. Chiaramoni N. S. Alonso S. D. V. Prieto M. J. (2017). Nanotoxicological and Teratogenic Effects: A Linkage between Dendrimer Surface Charge and Zebrafish Developmental Stages. Toxicol. Appl. Pharmacol. 337, 1–11. 10.1016/j.taap.2017.10.003 28993268

[B12] Courtney P. A. Crockard A. D. Williamson K. Irvine A. E. Kennedy R. J. Bell A. L. (1999). Increased Apoptotic Peripheral Blood Neutrophils in Systemic Lupus Erythematosus: Relations with Disease Activity, Antibodies to Double Stranded DNA, and Neutropenia. Ann. Rheum. Dis. 58, 309–314. 10.1136/ard.58.5.309 10225817 PMC1752888

[B13] Davidson A. Diamond B. (2001). Autoimmune Diseases. N. Engl. J. Med. 345, 340–350. 10.1056/NEJM200108023450506 11484692

[B14] Davis M. E. Zuckerman J. E. Choi C. H. Seligson D. Tolcher A. Alabi C. A. (2010). Evidence of RNAi in Humans from Systemically Administered siRNA via Targeted Nanoparticles. Nature 464, 1067–1070. 10.1038/nature08956 20305636 PMC2855406

[B15] Dawulieti J. Sun M. Zhao Y. Shao D. Yan H. Lao Y. H. (2020). Treatment of Severe Sepsis with Nanoparticulate Cell-free DNA Scavengers. Sci. Adv. 6, eaay7148. 10.1126/sciadv.aay7148 32523983 PMC7259927

[B16] Deshpande M. C. Garnett M. C. Vamvakaki M. Bailey L. Armes S. P. Stolnik S. (2002). Influence of Polymer Architecture on the Structure of Complexes Formed by PEG-Tertiary Amine Methacrylate Copolymers and Phosphorothioate Oligonucleotide. J. Control. Release 81, 185–199. 10.1016/s0168-3659(02)00052-4 11992691

[B17] Dong C. Liu Y. Sun C. Liang H. Dai L. Shen J. (2020). Identification of Specific Joint-Inflammatogenic Cell-free DNA Molecules from Synovial Fluids of Patients with Rheumatoid Arthritis. Front. Immunol. 11, 662. 10.3389/fimmu.2020.00662 32411129 PMC7198838

[B18] Duvvuri B. Lood C. (2019). Cell-Free DNA as a Biomarker in Autoimmune Rheumatic Diseases. Front. Immunol. 10, 502. 10.3389/fimmu.2019.00502 30941136 PMC6433826

[B19] Dzmitruk V. Apartsin E. Ihnatsyeu-Kachan A. Abashkin V. Shcharbin D. Bryszewska M. (2018). Dendrimers Show Promise for siRNA and microRNA Therapeutics. Pharmaceutics 10. 10.3390/pharmaceutics10030126 PMC6161126 30096839

[B20] Fillatreau S. Manfroi B. Dörner T. (2021). Toll-like Receptor Signalling in B Cells during Systemic Lupus Erythematosus. Nat. Rev. Rheumatol. 17, 98–108. 10.1038/s41584-020-00544-4 33339987 PMC7747191

[B21] Fröhlich E. (2012). The Role of Surface Charge in Cellular Uptake and Cytotoxicity of Medical Nanoparticles. Int. J. Nanomedicine 7, 5577–5591. 10.2147/IJN.S36111 23144561 PMC3493258

[B22] Heidel J. D. (2011). Cyclodextrin-containing Polycations for Nucleic Acid Delivery. Cold Spring Harb Protoc. 2011, 1319–1322. 10.1101/pdb.top066639 22046045

[B23] Heidel J. D. Yu Z. Liu J. Y. Rele S. M. Liang Y. Zeidan R. K. (2007). Administration in Non-human Primates of Escalating Intravenous Doses of Targeted Nanoparticles Containing Ribonucleotide Reductase Subunit M2 siRNA. Proc. Natl. Acad. Sci. U S A. 104, 5715–5721. 10.1073/pnas.0701458104 17379663 PMC1829492

[B24] Holl E. K. Frazier V. Landa K. Boczkowski D. Sullenger B. Nair S. K. (2021). Controlling Cancer-Induced Inflammation with a Nucleic Acid Scavenger Prevents Lung Metastasis in Murine Models of Breast Cancer. Mol. Ther. 29, 1772–1781. 10.1016/j.ymthe.2020.12.026 33348055 PMC8116567

[B25] Holl E. K. Shumansky K. L. Borst L. B. Burnette A. D. Sample C. J. Ramsburg E. A. (2016). Scavenging Nucleic Acid Debris to Combat Autoimmunity and Infectious Disease. Proc. Natl. Acad. Sci. U S A. 113, 9728–9733. 10.1073/pnas.1607011113 27528673 PMC5024580

[B26] Holl E. K. Shumansky K. L. Pitoc G. Ramsburg E. Sullenger B. A. (2013). Nucleic Acid Scavenging Polymers Inhibit Extracellular DNA-Mediated Innate Immune Activation without Inhibiting Anti-viral Responses. PLoS One 8, e69413. 10.1371/journal.pone.0069413 23936008 PMC3720614

[B27] Hu C. M. Fang R. H. Luk B. T. Zhang L. (2014). Polymeric Nanotherapeutics: Clinical Development and Advances in Stealth Functionalization Strategies. Nanoscale 6, 65–75. 10.1039/c3nr05444f 24280870

[B28] Huang D. Qian H. Qiao H. Chen W. Feijen J. Zhong Z. (2018). Bioresponsive Functional Nanogels as an Emerging Platform for Cancer Therapy. Expert Opin. Drug Deliv. 15, 703–716. 10.1080/17425247.2018.1497607 29976103

[B29] Hwang S. J. Bellocq N. C. Davis M. E. (2001). Effects of Structure of Beta-Cyclodextrin-Containing Polymers on Gene Delivery. Bioconjug. Chem. 12, 280–290. 10.1021/bc0001084 11312690

[B30] Hwang T. L. Aljuffali I. A. Lin C. F. Chang Y. T. Fang J. Y. (2015). Cationic Additives in Nanosystems Activate Cytotoxicity and Inflammatory Response of Human Neutrophils: Lipid Nanoparticles versus Polymeric Nanoparticles. Int. J. Nanomedicine 10, 371–385. 10.2147/IJN.S73017 25609950 PMC4294622

[B31] Jain K. Kesharwani P. Gupta U. Jain N. K. (2010). Dendrimer Toxicity: Let's Meet the challenge. Int. J. Pharm. 394, 122–142. 10.1016/j.ijpharm.2010.04.027 20433913

[B32] Jain S. Pitoc G. A. Holl E. K. Zhang Y. Borst L. Leong K. W. (2012). Nucleic Acid Scavengers Inhibit Thrombosis without Increasing Bleeding. Proc. Natl. Acad. Sci. U S A. 109, 12938–12943. 10.1073/pnas.1204928109 22837404 PMC3420207

[B33] Jensen L. B. Pavan G. M. Kasimova M. R. Rutherford S. Danani A. Nielsen H. M. (2011). Elucidating the Molecular Mechanism of PAMAM-siRNA Dendriplex Self-Assembly: Effect of Dendrimer Charge Density. Int. J. Pharm. 416, 410–418. 10.1016/j.ijpharm.2011.03.015 21419201

[B34] Jiang X. Dai H. Ke C. Y. Mo X. Torbenson M. S. Li Z. (2007). PEG-b-PPA/DNA Micelles Improve Transgene Expression in Rat Liver through Intrabiliary Infusion. J. Control. Release 122, 297–304. 10.1016/j.jconrel.2007.06.014 17640758 PMC2035949

[B35] Karabasz A. Szczepanowicz K. Cierniak A. Bereta J. Bzowska M. (2018). *In Vitro* toxicity Studies of Biodegradable, Polyelectrolyte Nanocapsules. Int. J. Nanomedicine 13, 5159–5172. 10.2147/IJN.S169120 30233178 PMC6135212

[B36] Karabasz A. Szczepanowicz K. Cierniak A. Mezyk-Kopec R. Dyduch G. Szczęch M. (2019). *In Vivo* Studies on Pharmacokinetics, Toxicity and Immunogenicity of Polyelectrolyte Nanocapsules Functionalized with Two Different Polymers: Poly-L-Glutamic Acid or PEG. Int. J. Nanomedicine 14, 9587–9602. 10.2147/IJN.S230865 31824153 PMC6901045

[B37] Kawai S. Nishizawa M. (1984). New Procedure for DNA Transfection with Polycation and Dimethyl Sulfoxide. Mol. Cel Biol 4, 1172–1174. 10.1128/mcb.4.6.1172 PMC368888 6330534

[B38] Kim C. S. Nguyen H. D. Ignacio R. M. Kim J. H. Cho H. C. Maeng E. H. (2014). Immunotoxicity of Zinc Oxide Nanoparticles with Different Size and Electrostatic Charge. Int. J. Nanomedicine 9 Suppl 2 (Suppl. 2), 195–205. 10.2147/IJN.S57935 25565837 PMC4279726

[B39] Kubiritova Z. Radvanszky J. Gardlik R. (2019). Cell-Free Nucleic Acids and Their Emerging Role in the Pathogenesis and Clinical Management of Inflammatory Bowel Disease. Int. J. Mol. Sci. 20. 10.3390/ijms20153662 PMC6696129 31357438

[B40] Lee J. Sohn J. W. Zhang Y. Leong K. W. Pisetsky D. Sullenger B. A. (2011). Nucleic Acid-Binding Polymers as Anti-inflammatory Agents. Proc. Natl. Acad. Sci. U S A. 108, 14055–14060. 10.1073/pnas.1105777108 21844380 PMC3161575

[B41] Liang H. Peng B. Dong C. Liu L. Mao J. Wei S. (2018a). Cationic Nanoparticle as an Inhibitor of Cell-free DNA-Induced Inflammation. Nat. Commun. 9, 4291. 10.1038/s41467-018-06603-5 30327464 PMC6191420

[B42] Liang H. Yan Y. Wu J. Ge X. Wei L. Liu L. (2020). Topical Nanoparticles Interfering with the DNA-LL37 Complex to Alleviate Psoriatic Inflammation in Mice and Monkeys. Sci. Adv. 6, eabb5274. 10.1126/sciadv.abb5274 32923608 PMC7457336

[B43] Liang X. Liu L. Wei Y. Q. Gao G. P. Wei X. W. (2018b). Clinical Evaluations of Toxicity and Efficacy of Nanoparticle-Mediated Gene Therapy. Hum. Gene Ther. 29, 1227–1234. 10.1089/hum.2018.069 29893153 PMC6909678

[B44] Liu F. Sheng S. Shao D. Xiao Y. Zhong Y. Zhou J. (2021). A Cationic Metal-Organic Framework to Scavenge Cell-free DNA for Severe Sepsis Management. Nano Lett. 21, 2461–2469. 10.1021/acs.nanolett.0c04759 33686851 PMC8320336

[B45] Liu L. Liu Y. Xu B. Liu C. Jia Y. Liu T. (2018). Negative Regulation of Cationic Nanoparticle-Induced Inflammatory Toxicity through the Increased Production of Prostaglandin E2 via Mitochondrial DNA-Activated Ly6C+ Monocytes. Theranostics 8, 3138–3152. 10.7150/thno.21693 29896308 PMC5996362

[B46] Liu X. Liang H. Yan Y. Wu J. Bottini M. Liu X. (2021). The Protein corona Modulates the Inflammation Inhibition by Cationic Nanoparticles via Cell-free DNA Scavenging. Bioactive Mater. 10.1016/j.bioactmat.2021.10.044 PMC8843952 35224306

[B47] Mandel P. Metais P. (1948). Nuclear Acids in Human Blood Plasma. C R. Seances Soc. Biol. Fil 142, 241–243. 18875018

[B48] Mariappan N. Husain M. Zafar I. Singh V. Smithson K. G. Crowe D. R. (2020). Extracellular Nucleic Acid Scavenging Rescues Rats from Sulfur Mustard Analog-Induced Lung Injury and Mortality. Arch. Toxicol. 94, 1321–1334. 10.1007/s00204-020-02699-1 32157350 PMC7230031

[B49] Mastrobattista E. Hennink W. E. (2011). Polymers for Gene Delivery: Charged for success. Nat. Mater. 11, 10–12. 10.1038/nmat3209 22169909

[B50] McConnell K. I. Shamsudeen S. Meraz I. M. Mahadevan T. S. Ziemys A. Rees P. (2016). Reduced Cationic Nanoparticle Cytotoxicity Based on Serum Masking of Surface Potential. J. Biomed. Nanotechnol 12, 154–164. 10.1166/jbn.2016.2134 27301181 PMC4970519

[B51] Mecke A. Majoros I. J. Patri A. K. Baker J. R. Jr. Holl M. M. Orr B. G. (2005). Lipid Bilayer Disruption by Polycationic Polymers: the Roles of Size and Chemical Functional Group. Langmuir 21, 10348–10354. 10.1021/la050629l 16262291

[B52] Menjoge A. R. Kannan R. M. Tomalia D. A. (2010). Dendrimer-based Drug and Imaging Conjugates: Design Considerations for Nanomedical Applications. Drug Discov. Today 15, 171–185. 10.1016/j.drudis.2010.01.009 20116448

[B53] Miller S. D. Turley D. M. Podojil J. R. (2007). Antigen-specific Tolerance Strategies for the Prevention and Treatment of Autoimmune Disease. Nat. Rev. Immunol. 7, 665–677. 10.1038/nri2153 17690713

[B54] Mulens-Arias V. Rojas J. M. Pérez-Yagüe S. Morales M. P. Barber D. F. (2015). Polyethylenimine-coated SPIONs Trigger Macrophage Activation through TLR-4 Signaling and ROS Production and Modulate Podosome Dynamics. Biomaterials 52, 494–506. 10.1016/j.biomaterials.2015.02.068 25818455

[B55] Muñoz L. E. Lauber K. Schiller M. Manfredi A. A. Herrmann M. (2010). The Role of Defective Clearance of Apoptotic Cells in Systemic Autoimmunity. Nat. Rev. Rheumatol. 6, 280–289. 10.1038/nrrheum.2010.46 20431553

[B56] Naqvi I. Gunaratne R. McDade J. E. Moreno A. Rempel R. E. Rouse D. C. (2018). Polymer-Mediated Inhibition of Pro-invasive Nucleic Acid DAMPs and Microvesicles Limits Pancreatic Cancer Metastasis. Mol. Ther. 26, 1020–1031. 10.1016/j.ymthe.2018.02.018 29550075 PMC6079560

[B57] Pai M. Crowther M. A. (2012). Neutralization of Heparin Activity. Handb Exp. Pharmacol. 207, 265–277. 10.1007/978-3-642-23056-1_11 22566228

[B58] Palmerston Mendes L. Pan J. Torchilin V. P. (2017). Dendrimers as Nanocarriers for Nucleic Acid and Drug Delivery in Cancer Therapy. Molecules 22. 10.3390/molecules22091401 PMC5600151 28832535

[B59] Pannuzzo M. Esposito S. Wu L. P. Key J. Aryal S. Celia C. (2020). Overcoming Nanoparticle-Mediated Complement Activation by Surface PEG Pairing. Nano Lett. 20, 4312–4321. 10.1021/acs.nanolett.0c01011 32259451

[B60] Patil M. L. Zhang M. Minko T. (2011). Multifunctional Triblock Nanocarrier (PAMAM-PEG-PLL) for the Efficient Intracellular siRNA Delivery and Gene Silencing. ACS Nano 5, 1877–1887. 10.1021/nn102711d 21322531 PMC3062392

[B61] Peng B. Liang H. Li Y. Dong C. Shen J. Mao H. Q. (2019). Tuned Cationic Dendronized Polymer: Molecular Scavenger for Rheumatoid Arthritis Treatment. Angew. Chem. Int. Ed. Engl. 58, 4254–4258. 10.1002/anie.201813362 30724436

[B62] Pereira M. P. de Gomes M. G. Izoton J. C. Nakama K. A. Dos Santos R. B. Pinto Savall A. S. (2019). Cationic and Anionic Unloaded Polymeric Nanocapsules: Toxicological Evaluation in Rats Shows Low Toxicity. Biomed. Pharmacother. 116, 109014. 10.1016/j.biopha.2019.109014 31146108

[B63] Perret P. Bacot S. Gèze A. Gentil Dit Maurin A. Debiossat M. Soubies A. (2018). Biodistribution and Preliminary Toxicity Studies of Nanoparticles Made of Biotransesterified β-cyclodextrins and PEGylated Phospholipids. Mater. Sci. Eng. C Mater. Biol. Appl. 85, 7–17. 10.1016/j.msec.2017.12.017 29407159

[B64] Pham C. T. Mitchell L. M. Huang J. L. Lubniewski C. M. Schall O. F. Killgore J. K. (2011). Variable Antibody-dependent Activation of Complement by Functionalized Phospholipid Nanoparticle Surfaces. J. Biol. Chem. 286, 123–130. 10.1074/jbc.M110.180760 21047788 PMC3012966

[B65] Quadir M. A. Haag R. (2012). Biofunctional Nanosystems Based on Dendritic Polymers. J. Control. Release 161, 484–495. 10.1016/j.jconrel.2011.12.040 22245685

[B66] Rajasekaran D. Srivastava J. Ebeid K. Gredler R. Akiel M. Jariwala N. (2015). Combination of Nanoparticle-Delivered siRNA for Astrocyte Elevated Gene-1 (AEG-1) and All-Trans Retinoic Acid (ATRA): An Effective Therapeutic Strategy for Hepatocellular Carcinoma (HCC). Bioconjug. Chem. 26, 1651–1661. 10.1021/acs.bioconjchem.5b00254 26079152 PMC4783168

[B67] Reineke T. M. Davis M. E. (2003). Structural Effects of Carbohydrate-Containing Polycations on Gene Delivery. 1. Carbohydrate Size and its Distance from Charge Centers. Bioconjug. Chem. 14, 247–254. 10.1021/bc025592k 12526715

[B68] Ren Y. Jiang X. Pan D. Mao H. Q. (2010). Charge Density and Molecular Weight of Polyphosphoramidate Gene Carrier Are Key Parameters Influencing its DNA Compaction Ability and Transfection Efficiency. Biomacromolecules 11, 3432–3439. 10.1021/bm1009574 21067136 PMC3021761

[B69] Reyes-Reveles J. Sedaghat-Herati R. Gilley D. R. Schaeffer A. M. Ghosh K. C. Greene T. D. (2013). mPEG-PAMAM-G4 Nucleic Acid Nanocomplexes: Enhanced Stability, RNase protection, and Activity of Splice Switching Oligomer and Poly I:C RNA. Biomacromolecules 14, 4108–4115. 10.1021/bm4012425 24164501 PMC4295786

[B70] Rezaei R. Safaei M. Mozaffari H. R. Moradpoor H. Karami S. Golshah A. (2019). The Role of Nanomaterials in the Treatment of Diseases and Their Effects on the Immune System. Open Access Maced J. Med. Sci. 7, 1884–1890. 10.3889/oamjms.2019.486 31316678 PMC6614262

[B71] Rosenblum M. D. Remedios K. A. Abbas A. K. (2015). Mechanisms of Human Autoimmunity. J. Clin. Invest. 125, 2228–2233. 10.1172/JCI78088 25893595 PMC4518692

[B72] Rudin C. M. Marshall J. L. Huang C. H. Kindler H. L. Zhang C. Kumar D. (2004). Delivery of a Liposomal C-Raf-1 Antisense Oligonucleotide by Weekly Bolus Dosing in Patients with Advanced Solid Tumors: a Phase I Study. Clin. Cancer Res. 10, 7244–7251. 10.1158/1078-0432.CCR-04-0642 15534098

[B73] Rungsardthong U. Ehtezazi T. Bailey L. Armes S. P. Garnett M. C. Stolnik S. (2003). Effect of Polymer Ionization on the Interaction with DNA in Nonviral Gene Delivery Systems. Biomacromolecules 4, 683–690. 10.1021/bm025736y 12741785

[B74] Samal S. K. Dash M. Van Vlierberghe S. Kaplan D. L. Chiellini E. van Blitterswijk C. (2012). Cationic Polymers and Their Therapeutic Potential. Chem. Soc. Rev. 41, 7147–7194. 10.1039/c2cs35094g 22885409

[B75] Senzer N. Nemunaitis J. Nemunaitis D. Bedell C. Edelman G. Barve M. (2013). Phase I Study of a Systemically Delivered P53 Nanoparticle in Advanced Solid Tumors. Mol. Ther. 21, 1096–1103. 10.1038/mt.2013.32 23609015 PMC3666630

[B76] Stearns N. A. Lee J. Leong K. W. Sullenger B. A. Pisetsky D. S. (2012). The Inhibition of Anti-DNA Binding to DNA by Nucleic Acid Binding Polymers. PLoS One 7, e40862. 10.1371/journal.pone.0040862 22808279 PMC3394750

[B77] Sun T. M. Du J. Z. Yan L. F. Mao H. Q. Wang J. (2008). Self-assembled Biodegradable Micellar Nanoparticles of Amphiphilic and Cationic Block Copolymer for siRNA Delivery. Biomaterials 29, 4348–4355. 10.1016/j.biomaterials.2008.07.036 18715636

[B78] Sun Y. Guo F. Zou Z. Li C. Hong X. Zhao Y. (2015). Cationic Nanoparticles Directly Bind Angiotensin-Converting Enzyme 2 and Induce Acute Lung Injury in Mice. Part. Fibre Toxicol. 12, 4. 10.1186/s12989-015-0080-x 25890286 PMC4395934

[B79] Sun Y. Jiao Y. Wang Y. Lu D. Yang W. (2014). The Strategy to Improve Gene Transfection Efficiency and Biocompatibility of Hyperbranched PAMAM with the Cooperation of PEGylated Hyperbranched PAMAM. Int. J. Pharm. 465, 112–119. 10.1016/j.ijpharm.2014.02.018 24530382

[B80] Tan J. F. Too H. P. Hatton T. A. Tam K. C. (2006). Aggregation Behavior and Thermodynamics of Binding between Poly(ethylene Oxide)-Block-Poly(2-(diethylamino)ethyl Methacrylate) and Plasmid DNA. Langmuir 22, 3744–3750. 10.1021/la052591i 16584251

[B81] Tomalia D. A. Baker H. Dewald J. Hall M. Kallos G. Martin S. (1985). A New Class of Polymers: Starburst-Dendritic Macromolecules. Polym. J. 17, 117–132. 10.1295/polymj.17.117

[B82] Toy R. Pradhan P. Ramesh V. Di Paolo N. C. Lash B. Liu J. (2019). Modification of Primary Amines to Higher Order Amines Reduces *In Vivo* Hematological and Immunotoxicity of Cationic Nanocarriers through TLR4 and Complement Pathways. Biomaterials 225, 119512. 10.1016/j.biomaterials.2019.119512 31585233

[B83] Tug S. Helmig S. Menke J. Zahn D. Kubiak T. Schwarting A. (2014). Correlation between Cell Free DNA Levels and Medical Evaluation of Disease Progression in Systemic Lupus Erythematosus Patients. Cell Immunol 292, 32–39. 10.1016/j.cellimm.2014.08.002 25243646

[B84] Wang J. Gao S. J. Zhang P. C. Wang S. Mao H. Q. Leong K. W. (2004). Polyphosphoramidate Gene Carriers: Effect of Charge Group on Gene Transfer Efficiency. Gene Ther. 11, 1001–1010. 10.1038/sj.gt.3302248 14985789

[B85] Wei X. Shao B. He Z. Ye T. Luo M. Sang Y. (2015). Cationic Nanocarriers Induce Cell Necrosis through Impairment of Na(+)/K(+)-ATPase and Cause Subsequent Inflammatory Response. Cell Res 25, 237–253. 10.1038/cr.2015.9 25613571 PMC4650577

[B86] Wu J. J. Liang H. Y. Li Y. C. Shi Y. Bottini M. Chen Y. (2020a). Cationic Block Copolymer Nanoparticles with Tunable DNA Affinity for Treating Rheumatoid Arthritis. Adv. Funct. Mater. 30. 10.1002/adfm.202000391

[B87] Wu L. P. Ficker M. Christensen J. B. Trohopoulos P. N. Moghimi S. M. (2015). Dendrimers in Medicine: Therapeutic Concepts and Pharmaceutical Challenges. Bioconjug. Chem. 26, 1198–1211. 10.1021/acs.bioconjchem.5b00031 25654320

[B88] Wu L. P. Wang D. Li Z. (2020b). Grand Challenges in Nanomedicine. Mater. Sci. Eng. C Mater. Biol. Appl. 106, 110302. 10.1016/j.msec.2019.110302 31753337

[B89] Yamagata M. Kawano T. Shiba K. Mori T. Katayama Y. Niidome T. (2007). Structural Advantage of Dendritic poly(L-Lysine) for Gene Delivery into Cells. Bioorg. Med. Chem. 15, 526–532. 10.1016/j.bmc.2006.09.033 17035030

[B90] Yan Y. Liang H. Liu X. Liu L. Chen Y. (2021). Topical Cationic Hairy Particles Targeting Cell Free DNA in Dermis Enhance Treatment of Psoriasis. Biomaterials 276, 121027. 10.1016/j.biomaterials.2021.121027 34293700

[B91] Yonezawa S. Koide H. Asai T. (2020). Recent Advances in siRNA Delivery Mediated by Lipid-Based Nanoparticles. Adv. Drug Deliv. Rev. 154-155, 64–78. 10.1016/j.addr.2020.07.022 32768564 PMC7406478

[B92] Zhang P. Liu W. Peng Y. Han B. Yang Y. (2014). Toll like Receptor 4 (TLR4) Mediates the Stimulating Activities of Chitosan Oligosaccharide on Macrophages. Int. Immunopharmacol 23, 254–261. 10.1016/j.intimp.2014.09.007 25237008

[B93] Zhang P. C. Wang J. Leong K. W. Mao H. Q. (2005a). Ternary Complexes Comprising Polyphosphoramidate Gene Carriers with Different Types of Charge Groups Improve Transfection Efficiency. Biomacromolecules 6, 54–60. 10.1021/bm040010i 15638504

[B94] Zhang X. Q. Wang X. L. Zhang P. C. Liu Z. L. Zhuo R. X. Mao H. Q. (2005b). Galactosylated Ternary DNA/polyphosphoramidate Nanoparticles Mediate High Gene Transfection Efficiency in Hepatocytes. J. Control. Release 102, 749–763. 10.1016/j.jconrel.2004.10.024 15681095

[B95] Zhou J. Liu J. Cheng C. J. Patel T. R. Weller C. E. Piepmeier J. M. (2011). Biodegradable Poly(amine-Co-Ester) Terpolymers for Targeted Gene Delivery. Nat. Mater. 11, 82–90. 10.1038/nmat3187 22138789 PMC4180913

[B96] Zhu C. Jung S. Luo S. Meng F. Zhu X. Park T. G. (2010). Co-delivery of siRNA and Paclitaxel into Cancer Cells by Biodegradable Cationic Micelles Based on PDMAEMA-PCL-PDMAEMA Triblock Copolymers. Biomaterials 31, 2408–2416. 10.1016/j.biomaterials.2009.11.077 19963269

[B97] Zuckerman J. E. Gritli I. Tolcher A. Heidel J. D. Lim D. Morgan R. (2014). Correlating Animal and Human Phase Ia/Ib Clinical Data with CALAA-01, a Targeted, Polymer-Based Nanoparticle Containing siRNA. Proc. Natl. Acad. Sci. U S A. 111, 11449–11454. 10.1073/pnas.1411393111 25049380 PMC4128111

